# A Longitudinal Randomized Controlled Trial Protocol to Evaluate the Effects of Wuqinxi on Dynamic Functional Connectivity in Parkinson’s Disease Patients

**DOI:** 10.3389/fnhum.2021.711703

**Published:** 2021-09-10

**Authors:** Yuting Li, Lanlan Zhang, Yin Wu, Jian Zhang, Ke Liu

**Affiliations:** ^1^School of Nursing, Anhui University of Chinese Medicine, Hefei, China; ^2^School of Psychology, Shanghai University of Sport, Shanghai, China; ^3^School of Leisure Sport and Management, Guangzhou Sport University, Guangzhou, China; ^4^School of Economics and Management, Shanghai University of Sport, Shanghai, China; ^5^Shanghai Punan Hospital of Pudong New District, Shanghai, China

**Keywords:** Parkinson’s disease, Wuqinxi, intervention, cognition, dynamic functional connectivity

## Abstract

**Background:** Parkinson’s disease (PD) is a neurodegenerative movement disease that includes non-motor symptoms such as cognitive impairment. Long-term mind-body exercise has been shown to improve cognitive ability in PD patients, but the methods of assessment and intervention were inconsistent across studies. Wuqinxi is a mind-body exercise that is easy to learn, has few physical and cognitive demands, and is recommended for PD patients. Dynamic functional connectivity (DFC) has been associated with cognitive alterations in PD patients, but no studies have yet explored the effects of Wuqinxi on this association. The current protocol is designed to measure the effects of long-term Wuqinxi intervention on cognition in PD patients, and explore the underlying neural mechanisms through DFC.

**Methods:** A long-term single-blind, randomized trial will be conducted. PD patients and age- and gender-matched HC will be recruited; PD patients will be randomly assigned to either Wuqinxi or balance groups, and HC will all receive health education. The Wuqinxi group will receive a 90-min session of Wuqinxi intervention three times a week for 24 weeks, while the balance group will receive balance exercise instruction on the same schedule. Primary outcomes will include assessment of cognitive domains and dynamic temporal characteristics of functional connectivity. Secondary outcomes will include severity of motor symptoms, mobility, balance, and emotional state. Assessments will be conducted at baseline, at the end of 24 weeks of intervention, and 12 weeks after interventions have ended.

**Discussion:** This study will provide evidence to the effects of Wuqinxi exercise on cognitive improvements in PD patients from the perspective of DFC, and will contribute to the understanding of neural mechanisms underlying cognitive enhancement through Wuqinxi practice.

**Clinical Trial Registration:**www.chictr.org.cn, identifier ChiCTR2000038517.

## Introduction

Parkinson’s disease (PD) is a neurodegenerative disease characterized by motor impairment as well as non-motor symptoms such as cognitive impairment (Kim et al., 2017; Fiorenzato et al., 2019). Previous studies have shown that PD patients have cognitive deficits in executive, attentional, speech and language, visuospatial, and memory domains (Goldman and Sieg, 2020). Most interventional studies have focused on improving motor ability because it is the classical symptom of PD. Cognitive impairment, however, has not been addressed as thoroughly, even though it not only reduces quality of life for the patient but also increases cost of care and caregiver burden (Pigott et al., 2015). Cognitive impairment can occur from early to advanced stages of PD. Longitudinal studies have shown that more than 25% of patients with mild cognitive impairment develop dementia within 3 years and up to 50% of patients with early PD develop cognitive decline within 5 years (Broeders et al., 2013; Pedersen et al., 2013). Thus, the clinical management of cognitive impairment is a long-term challenge, and there is an urgent need to develop evidence-based treatments for cognitive enhancement in PD patients.

No medications have yet been shown to slow PD progression, but there is substantial evidence for long-term exercise attenuating PD progression (Ahlskog, 2018). A cross-sectional, questionnaire-based study showed that regular exercise was associated with less cognitive decline after 1 year (Oguh et al., 2014). There is growing evidence that mind-body exercise can improve cognition, executive function, learning, memory, and verbal fluency (Zhang Y. et al., 2018; Wu et al., 2019; Zhang et al., 2021). However, the methods for intervention and assessment were inconsistent across studies, so it is difficult to draw conclusions about the mechanisms of mind-body exercise on cognition. In order to clarify the mechanisms, we should first focus on only one approach and use a variety of tools to track and assess the effectiveness of the intervention.

Previous studies have shown that mind-body exercise can improve the cognitive abilities of middle-aged and elderly people (Zou et al., 2019). Wuqinxi imitates the movement and breathing patterns of five animals (tigers, deer, bears, apes, and birds) and emphasizes the integration of body, breath, and mind (Guo et al., 2018). It is a kind of mind-body exercise which is easy to learn and has few physical and cognitive demands (Guo et al., 2018; Zou et al., 2019). Thus, PD patients with physical and cognitive impairments may choose Wuqinxi as a complement to medicine (Zhang et al., 2014). However, few studies have been conducted to find the neural mechanisms underlying the improvements (Zhang et al., 2021). Thus, this long-term study protocol focuses on Wuqinxi and uses dynamic functional connectivity (DFC) to detect the cognitive changes in PD patients.

Studies have shown that temporal variation in functional connectivity during scanning time reflected cognitive changes in PD patients (Kim et al., 2017; Fiorenzato et al., 2019). Functional connectivity analysis is based on temporal correlations between spatially remote neurophysiological events (Friston, 1994; Di and Biswal, 2015). In the past, researchers have typically computed static functional connectivity (SFC) with the assumption that it remains constant throughout the duration of the scan (Friston, 1994; Di and Biswal, 2015). In recent years, however, a large number of researchers realized that functional connectivity in fact varies periodically (Chang and Glover, 2010; Allen et al., 2014; Di and Biswal, 2015). Compared with DFC, time averaged SFC ignore the underlying temporal variations of functional connectivity which may provide additional information about brain function (Zhang C. et al., 2018). This offers yet another dimension upon which to evaluate disease states, and DFC has since been applied to depression (Kaiser et al., 2016), Alzheimer’s disease (Schumacher et al., 2019), and Parkinson’s disease (Kim et al., 2017; Fiorenzato et al., 2019). Specifically, DFC was found abnormal at the early stage in PD and correlated with disease progression, cognitive impairments (Kim et al., 2017; Cordes et al., 2018; Díez-Cirarda et al., 2018).

The aim of this study protocol is to explore the effects of Wuqinxi intervention for cognitive impairment in PD by DFC analysis. We hypothesize that Wuqinxi will change the temporal characteristics of DFC as well as the motor and cognitive impairments of PD patients.

## Methods

The current study is designed as a longitudinal, single-blind, randomized controlled trial. The study has been approved by the local research ethics committee.

### Design and Procedures

**FIGURE 1:**
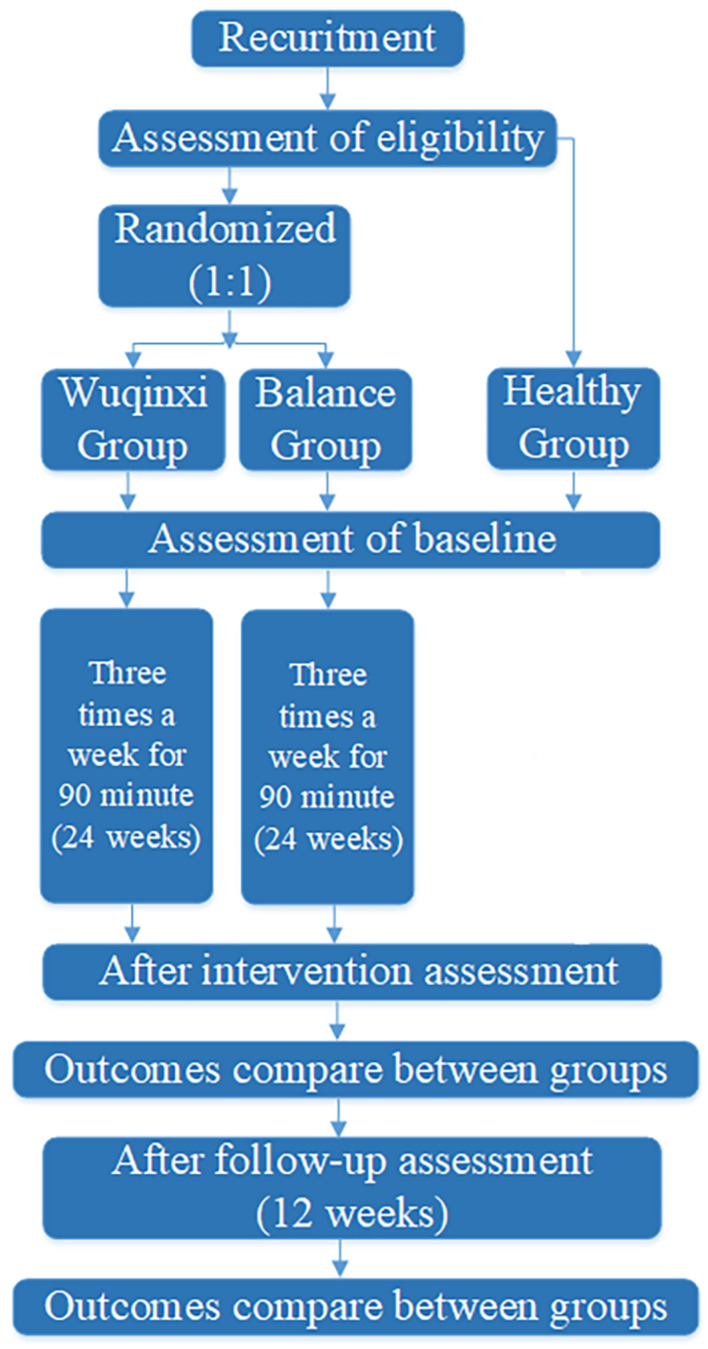
Study pipeline.

PD participants will be recruited through the local hospital’s exercise intervention group. To reduce potential expectation bias and confirm eligibility, a research assistant will make telephone contact with those referred by a neurologist and follow up with interested participants. Healthy controls (HC) will be recruited from the local communities through posters during the same recruitment period. Participants with PD will be randomly assigned to one of two groups: Wuqinxi or balance. The total intervention period will be 24 weeks. Primary and secondary outcomes will be assessed at baseline (prior to intervention), immediately following the end of the intervention period, and 12 weeks after the intervention has ended (see Figure 1). All PD participants will follow their regular medication scheme during the study period. This protocol has been approved by the Ethics Committee of Shanghai University of Sport (102772020RT107). All participants who meet the inclusion criteria will sign informed consent prior to the study.

A recently published protocol “Long-Term Wu Qin Xi Exercise on Response Inhibition and Cortical Connectivity in Parkinson’s Disease: Design and Implementation of a Randomized Controlled Clinical Trial” is similar to the current protocol (Wang Z. et al., 2021). Our protocol considers the whole brain and will use resting-state fMRI to detect the effects of Wuqinxi on PD patients, while the previously published one only take the primary motor cortex and response inhibition related brain regions (dorsal lateral prefrontal cortex, right inferior frontal gyrus, and presupplementary motor area) into consideration and use dual-site paired-pulse transcranial magnetic stimulation to evaluate the cortical connectivity.

We will first follow the methods in Fiorenzato and Kim’s researches to replicate the effects in their work (Kim et al., 2017; Fiorenzato et al., 2019), and then use DFC analysis as the main method to detect the effects of wuqinxin intervention in patients. In order to replicate the effects in Fiorenzato and Kim’s study, HC will be recruited and compared with whole PD patients before the intervention to support that DFC method could be used in our participants. HC will not be involved in the intervention procedure.

### Participants

#### Parkinson’s Disease Patients

##### Inclusion criteria

• Patients with PD diagnosed according to the UK Parkinson’s Disease Society Brain Bank diagnostic criteria.
• Age 55–75 years old.
• Hoehn and Yahr (H&Y) score of 1–3.
• 20≤ Montreal Cognitive Assessment (MoCA) score ≤25.
• Ability to walk and exercise independently.

##### Exclusion criteria

• Tai Chi or other exercise habits.
• Other diseases that could interfere with conduct of the study protocol.
• History of head injury.
• Other significant psychiatric, neurological, or systemic comorbidity.
• Magnetic Resonance Imaging (MRI) signal abnormalities (such as cerebral vascular lesions, white matter hyperintensities, and evidence of space-occupying lesions) or artifacts.
• Severe cognitive, visual, or auditory impairment.
• Depression or anxiety.
• Deep brain stimulation.
• MRI contraindications.
• Alcohol or drug dependency or abuse.

#### Healthy Controls

##### Inclusion criteria

• Age 55–75 years old.
• Ability to walk and exercise independently.

##### Exclusion criteria

• Tai Chi or other exercise habits.
• Other diseases that could interfere with conduct of the study protocol.
• History of head injury.
• Other significant psychiatric, neurological, or systemic comorbidity.
• MRI signal abnormalities (such as cerebral vascular lesions, white matter hyperintensities, and evidence of space-occupying lesions) or artifacts.
• Severe cognitive, visual, or auditory impairment.
• Depression or anxiety.
• MRI contraindications.
• Alcohol or drug dependency or abuse.

### Sample Size Calculation and Blinding

Sample size estimation was performed by G^∗^power V3.1 software (Franz Faul, Universitat Kiel, Germany). According to the analysis, at least 44 participants should be recruited (22 participants per group) in order to be able to perform two-factor repeated ANOVA (within-between interaction) with a middle effect size of 0.25 (Cohen, 1988), level of significance of 0.05, and power of 95% (Mayr et al., 2007). Since some participants may need to be dropped from the analysis due to excessive head motion during scanning, 40 participants will be recruited for each PD subgroup and 40 HC will be recruited. According to a similar studies published recently (Fiorenzato et al., 2019; Chen et al., 2021), this sample size should be adequate for replicating the previous effects in Fiorenzato’s study. In order to attract more participants, we will offer generous compensation and long-term health guidance. Every participant will be offered with an individual exercise plan when the whole study was completed. Randomization of PD patients will be done at the website www.random.org. Two researchers will be trained to assess the outcomes, and they will be blinded to group and will not participate in any interventions.

### Interventions

Participants in all PD patients’ groups will be asked to record their exercise every day throughout the trial, including both in the laboratory and at home.

#### Wuqinxi Training

**FIGURE 2:**
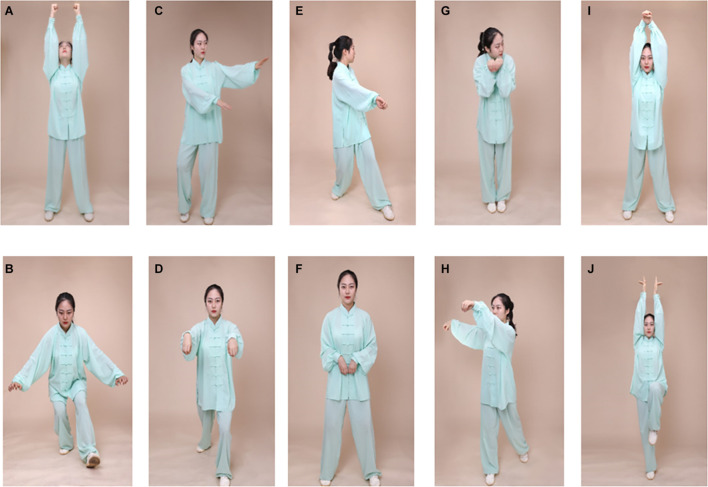
Illustration of Wuqinxi exercise maneuvers. **(A,B)** Tiger exercise; **(C,D)** deer exercise; **(E,F)** bear exercise; **(G,H)** monkey exercise; **(I,J)** bird exercise. Written informed consent for publication of the pictures was obtained from the poster demonstrator.

Participants in the Wuqinxi group will attend a 24-week Wuqinxi exercise program consisting of 90-min group sessions three times a week. Each session will begin with 10 min of warm-up consisting of a breathing exercise, slow walking, and range-of-motion exercises, followed by 60 min of core Wuqinxi exercise (see Figure 2) with a 10-min break in the middle and 10 min of cooldown session consisting of limb range-of-motion movements, sustained stretching, and relaxing. The warm-up will focus on breaking down the movements and getting familiar with the main points of the movements; the later three steps will focus on consistency of movement, fluency of gait, and balance of posture. Heart rates will be monitored throughout the session using Polar Team2 heart rate monitors (Polar Electro, Finland). The intensity and difficulty, timing and frequency of the exercise will be adjusted for each participant based on their heart rate. Participants will be encouraged to practice at home, but not to participate in other exercises during the 24-week intervention period.

#### Balance Training

Participants in the balance group will attend a 24-week balance training exercise program consisting of 90-min group sessions three times a week. Each training session will start with a 10 min warm-up (same as wuqinxi training). Then a 30-min balance exercise will be performed twice, with a 10-min break in between. Training will include the following sections: (1) static balance training: standing on unstable surfaces to maintain postural control and progression to weight shifting; (2) dynamic balance training: postural control in standing position while adding upper limb and trunk movements; (3) balance strategy exercise: focus on hip strategy while maintaining ankle strategy and stepping strategy under interference in different directions; and (4) adaptation of varying base of support, and standing in a narrow space and on uneven surface (Atterbury and Welman, 2017; Li et al., 2019). Each training session will end with a 10-min cooldown session (same as wuqinxi training).

#### Healthy Controls

Participants in the healthy control group will not receive any intervention exercise.

### Resting-State and Structural MRI Acquisition and Preprocessing

Resting state functional MRI data and 3D high-dimension T1 image will be collected using a 3T Siemens Magnetom Prisma MRI scanner. PD patients will take medication during MRI scanning to minimize movement artifacts and reduce discomfort. All participants will be instructed to keep their eyes open and focus on a white “+” on black screen, letting their minds wander and trying not to fall asleep during the 10 min 6 s resting state functional MRI scan (gradient echo/fast gradient echo pulse sequence: repetition time = 2000 ms; echo time = 30 ms; flip angle = 80°; matrix size = 106 × 106; number of slices = 62; and voxel size = 2.0 × 2.0 × 2.0 mm^3^ with no gap; number of total volume = 304). During the structural scan participants will be instructed to sleep (repetition time = 3130 ms; echo time = 2.98 ms; flip angle = 4°; matrix size = 256 × 256; number of slice = 176; voxel size = 1.0 × 1.0 × 1.0 mm^3^).

Data preprocessing will be conducted using the SPM12 package implemented in MATLAB R2019a^1^. Prior to preprocessing (1) the first four scans will be deleted from the resting functional volume; (2) functional scans will be realigned to the first volume to correct for interscan head motion; (3) functional scans will be segmented into gray matter, white matter, and cerebrospinal fluid using the Tissue Probability Map template; and finally, and (4) normalized into standard Montreal Neurological Institute space using non-linear transformations and spatially smoothed with a Gaussian smoothing kernel of 6 mm. Participants with head motion greater than 1 mm will be removed from the study (Liao et al., 2014).

### DFC Characteristics Computation

#### Group Independent Component Analysis

After preprocessing, the resting state data of all participants will be analyzed using spatial independent component analysis implemented in GIFT^2^ to decompose the data into functional networks that exhibit a unique time course profile.

Reduction steps will be carried out at the subject- and group-levels. Subject-specific data will be reduced to 120 components, then concatenated across time. Next, at the group level, data will be reduced to 100 independent components using the expectation-maximization algorithm. Reliability and stability of the infomax independent component analysis algorithm in ICASSO will be ensured by repeating the algorithm 20 times.

The resulting components will be clustered to estimate their reliability and components with values >0.80 will be saved. Subject-specific spatial maps and time courses will be obtained using the back-reconstruction approach.

#### Dynamic functional connectivity

##### Sliding Window Approach

Sliding window analysis will be applied using the DFC network toolbox in GIFT. Resting state data will be separated into windows of 22 repetition times (44 s), and the regularized inverse covariance matrix will be used as covariance estimation using shorter time series can be noisy. An additional L1 norm of the precision matrix will be imposed to promote sparsity in the graphic LASSO framework with 100 repetitions. Subsequently, the functional connectivity matrices will be transformed to z-scores using Fisher’s z-transformation and covariates (age, gender, and education) will be regressed out.

##### Clustering analysis

A K-means clustering algorithm will be applied to the windowed functional connectivity matrices to assess the recurring functional connectivity patterns (states), as expressed by the frequency and structure of these states. Then a cluster validity analysis will be performed on the exemplars of all subjects to estimate the optimal number of clusters. We will use a subset of windows (local maxima in functional connectivity variance) as subject exemplars to decrease the redundancy between windows and computational demands. The reproducibility of functional connectivity states will be established using bootstrap resampling and split-half sample methods. The optimal number of clusters will be set to *k* = 2 following previous studies which is determined based on the silhouette criterion of cluster validity index (Rousseeuw, 1987; Fiorenzato et al., 2019).

### Outcome Measures

All measurements will be conducted before the intervention begins (baseline), immediately after the 24 weeks of intervention, and 12 weeks after the intervention.

#### Participants’ Characteristics

Demographic characteristics of all participants will be assessed prior to intervention including age, gender, education, age of onset, disease duration, body mass (kg/m^2^), height (cm), and family situation (see Table 1). Levodopa equivalent daily dosages will be calculated for PD patients (Tomlinson et al., 2010).

**TABLE 1:** Demographic characteristics.

	Wuqinxi (*n*)	Balance (*n*)	HC (*n*)	Whole PD vs. HC (*t*/*x*^2^, *p*)	Wuqinxi vs. Balance (*t*/*x*^2^/*F*, *p*)*
Age (year)					
Gender (male%: female%)					
Education (year)					
Age of onset (year)				–	
Hoehn and Yahr stage, *n* (%)					
1–1.5				–	
2–2.5				–	
3				–	
Disease duration				–	
Body mass (Kg/m^2^)					
Height (cm)					
Family situation					
Living along					
Living with husband/wife					
Living with husband/wife and children					
LEDD				–	*

The Chi-square test will be used for categorical variables, and two-sample t-test will be run to test between group differences for continuous variables. LEDD = levodopa equivalent daily dose. *Two-factor repeated ANOVA will be applied.

#### Primary Outcome Assessment

The Montreal Cognitive Assessment (Nasreddine et al., 2005) will be conducted to assess cognitive impairment and the Repeatable Battery for the Assessment of Neuropsychological Status (Stella et al., 2013) will be used to measure the cognitive domains of immediate memory, visuospatial, language, attention, and delayed memory (see Supplementary Table 1). Dynamic states, dwell times, fractional windows, and number of transitions will be computed to reflect the characteristics of DFC (see Supplementary Table 1).

#### Secondary Outcome Assessments

The Parkinson’s Disease Sleep Scale (PDSS) will be used to measure the severity of sleep disturbances (Trenkwalder et al., 2011); Parkinson Disease Questionnaire (PDQ-39) will be used to assess PD-specific health quality over the previous month (Jenkinson et al., 1997); Timed Up and Go Test (TUGT) will be used to evaluate mobility and motor ability (Zampieri et al., 2010); Unified Parkinson’s Disease Rating Scale Part III (UPDRS-III) will be used to measure motor symptoms (Haaxma et al., 2008); Hamilton Depression Scale [HAMD] (Bech et al., 1981) and Hamilton Anxiety Scale[HAMA] (Shear et al., 2010) will be used to evaluate depression and anxiety in both PD patients and HC (see Supplementary Table 1).

### Statistical Analysis

Following a previous study (Fiorenzato et al., 2019), we will investigate the temporal properties of DFC states by computing the average dwell time and fractional windows in each state, as well as the number of transitions from one state to another.

#### Replication of the previous effects

##### Clinical and Neuropsychological Data Analysis

Statistical analyses will be performed using SPSS version 19.0. Demographic and clinical information of all PD patients will be compared using two-sample independent *t*-test (see Table 1). Pearson’s chi-square test will be used to compare categorical variables (see Table 1). Pearson’s correlation analyses will be applied between temporal properties and cognition (MoCA and cognitive performance in each domain) in whole PD patients as well as motor severity (UPDRS-III) before the intervention (see Table 2). The threshold for statistical significance will be set at *p* < 0.05.

**TABLE 2:** Correlation between dynamic functional connectivity temporal properties and clinical characteristics (before intervention, after intervention, and follow-up).

		UPDRS-III	MoCA	Cognitive domains				
				I-M	V	L	A	D-M
Dwell time state I	*r*							
	*p*-Value							
Dwell time state II	*r*							
	*p*-Value							
Fractional windows	*r*							
	*p*-Value							
Number of transitions	*r*							
	*p*-Value							

*I-M = immediate memory; V = visuospatial; L = language; A = attention; D-M = delayed memory.*

##### Group differences in dynamic connectivity: temporal and properties and strength

Group (whole PD patients vs. HC) differences in dwell time, fractional windows, and number of transitions will be examined using two-sample independent *t*-test analysis (*p* < 0.05, FDR correction, see Supplementary Table 1). Covariates will include education, levodopa and dopamine agonist equivalent daily doses. Subsequently, subject-specific medians corresponding to each group-level state will be estimated and two-sample independent *t*-test analysis will be used to compare the connectivity strength of each state at each unique regional pairing (*p* < 0.05, FDR correction) between groups (whole PD patients vs. HC), see Supplementary Table 1. Further, two-sample independent *t*-test analysis will be performed to compare strength abnormalities associated with cognitive deficits by comparing whole PD patients and HC (*p* < 0.05, FDR correction), see Supplementary Table 1.

#### Application of DFC in Wuqinxi Intervention

##### Clinical and neuropsychological data analysis

Statistical analyses will be performed using SPSS version 19.0. Demographic and clinical information of two PD subgroups (wuqinxi group vs. balance group) will be compared across the three timepoints (baseline, 24 weeks, and 12-week follow-up) using two-factor repeated analysis of variance (ANOVA), see Table 1. Pearson’s chi-square test will be used to compare categorical variables (see Table 1). Pearson’s correlation analyses will be applied between temporal properties and cognition (MoCA and cognitive performance in each domain) across two PD subgroups as well as motor severity (UPDRS-III) before the intervention (see Table 2). Then, the same correlation analyses will be used after the intervention and will also be carried out after follow-up (see Table 2). The threshold for statistical significance will be set at *p* < 0.05.

##### Group differences in dynamic connectivity: temporal and properties and strength

Between-group (wuqinxigroup vs. balance group) differences in dwell time, fractional windows, and number of transitions will be examined using two-factor repeated ANOVA (*p* < 0.05, FDR correction), see Supplementary Table 1. Covariates will include education, levodopa and dopamine agonist equivalent daily doses. Continuously, subject-specific medians corresponding to each group-level state will be estimated and two-factor repeated ANOVA will be used to compare the connectivity strength of each state at each unique regional pairing (*p* < 0.05, FDR correction) between the three timepoints (before intervention, after intervention, and follow-up), see Supplementary Table 1. Finally, two-factor repeated ANOVA will be performed to compare strength abnormalities associated with cognitive deficits by comparing different timepoints of Wuqinxi group and balance group (*p* < 0.05, FDR correction), see Supplementary Table 1.

## Discussion

To date, little is understood about the neural mechanisms underlying the improvement of cognitive ability in PD patients after mind-body exercise. Previous studies suggested that mind-body exercise can enhance cognitive performance in PD patients (Zhang Y. et al., 2018; Wu et al., 2019; Zhang et al., 2021). Among the various types of mind-body exercises, Wuqinxi has often been recommended as a complement to medicine to treat cognitive impairment (Zhang et al., 2014). Thus, the present study proposes a longitudinal protocol for enhancing cognition in PD patients with Wuqinxi intervention.

Previous studies have shown that dynamic temporal properties can reflect cognitive alterations in PD patients (Kim et al., 2017; Fiorenzato et al., 2019). Two distinct connectivity ‘states’ have been observed in PD patients: one is more frequent and characterized by a predominance of within-network connections, while the other is less frequent and characterized by between-network connections (Fiorenzato et al., 2019). The dwell time and transitions between these two states was significantly correlated with various cognitive domains in PD patients, suggesting that analysis of these states may help to further our understanding of neural mechanisms of cognitive enhancement. DFC provides a method with which to explore the effects of Wuqinxi at the brain network level, complementing the traditional evidence that focuses more on behavioral performance and motor function (Reynolds et al., 2016).

There are a few shortcomings and in this protocol that have to be considered. First, we calculated the simple size following the principles of behavior science (Cohen, 1988), but it may still be small for neuroscience. Second, we cannot exclude that dopaminergic therapy will have some effect on functional connectivity (Berman et al., 2016). Third, the depression/anxiety of patients may will also have effect on functional connectivity (Yan et al., 2020; Wang C. et al., 2021). Further, it is difficult to guarantee that after randomization wuqinxin- and balance-group is strict matched for baseline cognition.

## Ethics Statement

The studies involving human participants were reviewed and approved by the Ethics Committee of Shanghai University of Sport. The patients/participants provided their written informed consent to participate in this study. Written informed consent was obtained from the individual(s) for the publication of any potentially identifiable images or data included in this article.

## Author Contributions

YL: conceptualization, methodology, investigation, and Original draft writing. LZ: visualization, software, formal analysis, and validation. YW: data curation. JZ: writing review, editing, and project Administration. KL: conceptualization, supervision, and funding acquisition. All authors had read and approved the final manuscript.

## Conflict of Interest

The authors declare that the research was conducted in the absence of any commercial or financial relationships that could be construed as a potential conflict of interest.

## Publisher’s Note

All claims expressed in this article are solely those of the authors and do not necessarily represent those of their affiliated organizations, or those of the publisher, the editors and the reviewers. Any product that may be evaluated in this article, or claim that may be made by its manufacturer, is not guaranteed or endorsed by the publisher.

## Footnotes

1
https://www.fil.ion.ucl.ac.uk/spm/software/spm12/

2
http://icatb.sourceforge.net/groupica.htm

## Supplementary Material

Supplementary Table 1 Changes over time within and between groups.

## Abbreviations

PD: Parkinson’s Disease
HC: Healthy Controls
SFC: Static Functional Connectivity
DFC: Dynamic Functional Connectivity
MoCA: Montreal Cognitive Assessment
PDSS: The Parkinson’s Disease Sleep Scale
PDQ-39: Parkinson Disease Questionnaire
TUGT: Timed Up and Go Test
UPDRS-III: Unified Parkinson’s Disease Rating Scale Part III
HAMA: Hamilton Anxiety Scale
HAMD: Hamilton Depression Scale.
