# Supplementary material for: A Longitudinal Randomized Controlled Trial Protocol to Evaluate the Effects of Wuqinxi on Dynamic Functional Connectivity in Parkinson’s Disease Patients
Source: Front Hum Neurosci. 2021 Sep 10;15:711703. doi: 10.3389/fnhum.2021.711703 (PMC8461094; doi:10.3389/fnhum.2021.711703)
Supplement: Supplementary Table 1 — Changes over time within and between groups. [file Table_1.DOCX]

**Supplementary Table 1. Changes over time within and between groups**

|  | | | | | | | | | | |
| --- | --- | --- | --- | --- | --- | --- | --- | --- | --- | --- |
| **Group** | **Base-line** | **24 weeks** | **Follow**  **up** | **Mean difference at 12 weeks** | **Mean difference at follow-up** | **F (*p-*value)**  **Time effect** | | **F (*p-*value)**  **Group effect** | **Whole PD vs. HC**  **(t, p)** | **F (*p-*value) Interaction effect** |
| **Primary outcomes** | | | | | | | | | | |
| **MoCA** | | | | | | | | | | |
| Wuqinxi |  |  |  |  |  |  |  | |  |  |
| Balance |  |  |  |  |  |  |  |  |  |  |
| Healthy |  | - | - | - | - | - | - | |  | - |
| **Immediate Memory** | | | | | | | | | | |
| Wuqinxi |  |  |  |  |  |  |  | |  |  |
| Balance |  |  |  |  |  |  |  |  |  |  |
| Healthy |  | - | - | - | - | - | - | |  | - |
| **Visuospatial** | | | | | | | | | | |
| Wuqinxi |  |  |  |  |  |  |  | |  |  |
| Balance |  |  |  |  |  |  |  |  |  |  |
| Healthy |  | - | - | - | - | - | - | |  | - |
| **Language** | | | | | | | | | | |
| Wuqinxi |  |  |  |  |  |  |  | |  |  |
| Balance |  |  |  |  |  |  |  |  |  |  |
| Healthy |  | - | - | - | - | - | - | |  | - |
| **Attention** | | | | | | | | | | |
| Wuqinxi |  |  |  |  |  |  |  | |  |  |
| Balance |  |  |  |  |  |  |  |  |  |  |
| Healthy |  | - | - | - | - | - | - | |  | - |
| **Delayed Memory** | | | | | | | | | | |
| Wuqinxi |  |  |  |  |  |  |  | |  |  |
| Balance |  |  |  |  |  |  |  |  |  |  |
| Healthy |  | - | - | - | - | - | - | |  | - |
| **Dwell time state I** | | | | | | | | | | |
| Wuqinxi |  |  |  |  |  |  |  | |  |  |
| Balance |  |  |  |  |  |  |  |  |  |  |
| Healthy |  | - | - | - | - | - | - | |  | - |
| **Dwell time state II** | | | | | | | | |  |  |
| Wuqinxi |  |  |  |  |  |  |  | |  |  |
| Balance |  |  |  |  |  |  |  |  |  |  |
| Healthy |  | - | - | - | - | - | - | |  | - |
| **Fractional windows** | | | | | | | | | | |
| Wuqinxi |  |  |  |  |  |  |  | |  |  |
| Balance  Healthy |  |  |  |  |  |  |  |  |  |  |
|  |  | - | - | - | - | - | - | |  | - |
| **Number of transitions** | | | | | | | | | | |
| Wuqinxi |  |  |  |  |  |  |  | |  |  |
| Balance |  |  |  |  |  |  |  |  |  |  |
| Healthy |  | - | - | - | - | - | - | |  | - |
| **Secondary outcomes** | | | | | | | | | | |
| **PDSS** | | | | | | | | | |  |
| Wuqinxi |  |  |  |  |  |  |  | | - |  |
| Balance |  |  |  |  |  |  |  |  |  |  |
| **PDQ-39** | | | | | | | | | | |
| Wuqinxi |  |  |  |  |  |  |  | | - |  |
| Balance |  |  |  |  |  |  |  |  |  |  |
| **TUGT** | | | | | | | | | | |
| Wuqinxi |  |  |  |  |  |  |  | | - |  |
| Balance |  |  |  |  |  |  |  |  |  |  |
| **UPDRS-III** | | | | | | | | | | |
| Wuqinxi |  |  |  |  |  |  |  | | - |  |
| Balance |  |  |  |  |  |  |  |  |  |  |
| **HAMA** | | | | | | | | | | |
| Wuqinxi |  |  |  |  |  |  |  | |  |  |
| Balance |  |  |  |  |  |  |  |  |  |  |
| Healthy |  |  |  |  |  |  | - | |  | - |
| **HAMD** | | | | | | | | | | |
| Wuqinxi |  |  |  |  |  |  |  | |  |  |
| Balance |  |  |  |  |  |  |  |  |  |  |
| Healthy |  |  |  |  |  |  | - | |  | - |
